# Correlation analysis between visual factors and academic performance in Chinese children

**DOI:** 10.1038/s41598-026-46397-x

**Published:** 2026-04-01

**Authors:** Guoyong Liu, Bin Guo, Junli Yu, Qian Xu, Yirong Wang, Peiran Si, Yizhuo Gong, Huanhuan Huo, Xiaohui Ma, Yuanyuan Hu, Hongsheng Bi

**Affiliations:** 1 Affiliated Eye Hospital of Shandong University of Traditional Chinese Medicine, Jinan, China; 2 Ophthalmology & Optometry Medical School, Shandong University of Traditional Chinese Medicine, Jinan, China; 3 Women & Children’s Health Care Hosptial of Huantai, Zibo, China

**Keywords:** Visual acuity, Astigmatism, Ocular accommodation, Undercorrection, Academic performance, Diseases, Health care, Medical research

## Abstract

Vision plays a crucial role in children’s education, particularly the visual factors related to academic performance, which have been the subject of long-standing research. In a cross-sectional school-based study in Shandong Province, China, we assessed 1,766 primary school children aged 8.98 ± 1.23 years (grades 2–5) to identify key visual factors associated with academic performance. We assessed presenting visual acuity (PVA), astigmatism, binocular accommodative facility (AF) and amplitude of accommodation (AA). Myopia undercorrection was defined as PVA > 0.1 logMAR in myopic children, which could be improved by at least two lines using subjective refraction by increasing the minus lenses correction. Among the 26.1% (461/1,766) myopic children, 51.0% (235/461) were undercorrected. Compared to fully corrected children, those with undercorrected myopia scored significantly lower in Chinese (*P* = 0.011), Mathematics (*P* = 0.009), and average scores (*P* = 0.004). After adjusting for gender and intelligence quotient (IQ), multifactorial GLM analyses revealed that PVA (β = -7.03, 95% *CI*: -10.63 to -3.43; *P* < 0.001) and astigmatism (β = -1.44, 95% *CI*: -2.33 to -0.55; *P* = 0.001) were significantly negatively correlated with academic performance. In contrast, binocular AF (β = 0.16, 95% *CI*: 0.06 to 0.26;*P* = 0.002) and AA (β = 0.18, 95% *CI*༚0.05 to 0.30༛*P* = 0.006) were significantly positively correlated with academic performance. After excluding children with premyopia and hyperopia and adjusting for relevant factors, myopia undercorrection remained significantly negatively correlated with academic performance (β = -2.88, 95% *CI*: -4.68 to -1.09; *P* = 0.002). These results show that poorer visual acuity, higher astigmatism, reduced accommodative function, and myopia undercorrection are all associated with lower academic performance in children. Our findings support the implementation of regular visual acuity and functional assessments, as well as full correction of myopia in children.

## Introduction

In recent years, the global prevalence of myopia is rising and has become a significant public health concern, particularly threatening children’s ocular health^1^. This trend is significantly associated with prolonged educational near-work time, a known environmental risk factor for myopia in school-aged children^2^. More notably, myopia is associated with an elevated risk of ocular complications, potentially leading to long-term adverse health outcomes^3^. Within this context, conventional educational practices of extending study time to improve academic performance may pose risks to children’s visual health.

Visual acuity is crucial for learning, serving as an external indicator of the eye’s refractive status and visual function. Numerous studies have demonstrated a significant correlation between children’s visual acuity and academic performance^4–6^, wherein poor visual acuity can negatively impact performance. Other related visual factors, such as myopia, hyperopia, astigmatism, etc^7–12^., may also affect academic performance at different levels. However, existing studies largely focused on a single or several potential influencing factors without considering potential confounding effects or synergistic interactions among multiple visual factors.

Furthermore, evidence on the direct impact of binocular accommodative function, particularly accommodative facility (AF) and amplitude of accommodation (AA), on children’s academic performance remains notably scarce. Although a limited number of studies have assessed accommodative function, they have mainly focused on specific populations, such as children with reading difficulties^13^. Among Spanish children aged 6–12, Alvarez-Peregrina et al. found that those with poorer academic performance had both weaker distance visual acuity and a longer near point of convergence^11^. This finding suggests a potential association between accommodative function and academic performance. Therefore, large-scale studies are needed to systematically evaluate the relationship between accommodative function and academic performance in children.

Despite the availability of various myopia correction methods, undercorrection remains prevalent in clinical practice^14^. This suboptimal correction results in inadequate presenting visual acuity (PVA), potentially adversely affecting academic performance. However, systematic evaluation of this issue has been lacking in existing literature. Therefore, we conducted a school-based epidemiological study to comprehensively analyze visual factors associated with academic performance in children. And we hope this study can provide evidence for optimizing vision screening protocols in educational settings and promoting better academic performance.

## Methods

### Study participants

A school-based cross-sectional study was conducted in Huantai County, Shandong Province, China, between September and October 2021. Participants were recruited through a two-stage sampling approach. Firstly, two primary schools were randomly selected from the complete roster of county elementary schools. Subsequently, within each participating school, five classes per grade level (grades 2–5) were randomly selected using cluster sampling. All students in these selected classes were enrolled in the study. Children with strabismus, amblyopia, or other organic ocular pathologies were excluded from the study.

This study adhered to the principles of the Declaration of Helsinki and was approved by the Medical Ethics Committee of the Affiliated Eye Hospital of Shandong University of Traditional Chinese Medicine (No. HEC-KS-2020016KY01). Written informed consent was obtained from all children and their parents or legal guardians before participating in this study.

### Examination

Two ophthalmologists performed comprehensive and systematic slit-lamp and funduscopic examinations on all participants. Other assessments were performed by resident optometrists, including assessing PVA and best corrected visual acuity (BCVA, following subjective refraction) at a standard examination distance of 3 m (#600722, Good-Lite Co., Elgin, IL, USA), measuring intraocular pressure with noncontact tonometry (Topcon CT80; Topcon Corp., Tokyo, Japan), and testing non-cycloplegic and cycloplegic auto-refraction (Nidek ARK-1, Co., LTD, Japan). Additionally, the binocular accommodative facility (AF) and binocular amplitude of accommodation (AA) were measured. The inspection site adopted a uniform standard, and all instruments were calibrated daily before use. Refractive error measurements were performed thrice, with the average value recorded. The difference between the maximum and minimum values of the sphere and cylinder was required to be < 0.50 D, and the signal-to-noise ratio had to be ≥ 8; otherwise, remeasurement was needed.

Before the accommodative function tests, the test procedure was explained in detail to the children to ensure they understood the test content^15^. For children with uncorrected refractive errors, trial lens correction was provided based on their subjective refraction before AA and AF measurements. Under BCVA conditions, the children held a ± 2.00 D flipper and fixated on a 20/30 tumbling-E card (Baoshijia Technology Co., Ltd., Zhengzhou, China) at a test distance of 40 cm. The examiner recorded the number of cycles completed in 1 min. A valid cycle was defined as the child correctly identifying the orientation of the optotype (tumbling-E) through both the + 2.00 D and − 2.00 D lenses of the flipper. The result was expressed in cycles per minute (cpm). The average binocular AF was recorded after repeating the measurement at least thrice. Binocular AA was measured using the push-up method, where one end of the Accommodation Rule (OPT-IP-02, OPT-IP Technology Development Co., Ltd., Tianjin, China) was aligned with the tip of the child’s nose and the black-and-white near vision card placed horizontally at a distance of 40 cm. While the children were asked to fixate on the row of optotypes one line larger than their BCVA, the optometrist moved the card closer to the children at a rate of 1–2 cm per second until the optotype became persistently blurred, and the child reported immediately. This measurement was repeated thrice and the average value was recorded in diopters.

**Fig. 1:**
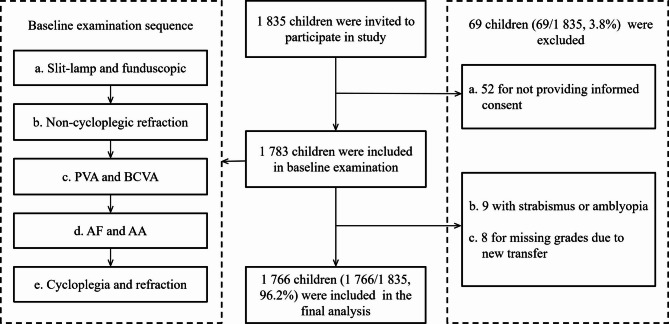
Flowchart of participant enrollment and examination procedures.

Cycloplegia was performed using 1% cyclopentolate hydrochloride solution (Alcon, Ft. Worth, Texas, USA). Three drops of 1% cyclopentolate were instilled in intervals of 5 min. Evaluation of pupil diameter and pupillary light reflex was performed approximately 30 min after the last drop was instilled. If the pupil was unresponsive to light and measured ≥ 6 mm in diameter, a auto-refractive assessment was performed^15^. Otherwise, an additional drop of cyclopentolate hydrochloride was administered, and followed by auto-refraction after 10 min. Other detailed steps of the ophthalmic examination have been reported in previous studies^16^. Figure 1 shows the flowchart for participant enrollment and examination procedures.

### Questionnaire and academic performance

Specialized researchers administered Raven’s Standard Progressive Matrices (SPM), and the children completed the test in the classroom for ≤ 40 min. The SPM is a non-literal Intelligence Quotient (IQ) test that does not require reading or language skills and assesses intelligence levels by discriminating the arrangement^17^. According to the correct number of SPM and age, age-specific IQ tertiles were obtained for the children [7 years old (tertile 1: ≤ 24, tertile 2: 25–43, tertile 3: ≥ 43), 8 years old (tertile 1: ≤ 30, tertile 2: 31–43, tertile 3: ≥ 44), 9 years old (tertile 1: ≤ 36, tertile 2: 37–46, tertile 3: ≥ 47), 10 years old (tertile 1: ≤ 40, tertile 2: 41–49, tertile 3: ≥ 50), 11 years old (tertile 1: ≤ 42, tertile 2: 43–51, tertile 3: ≥ 52)]^18^.

Academic performance was assessed as the mean score of the Chinese and mathematics final examinations from the spring semester of the 2020–2021 academic year. These examinations were administered and organized by the county-level education department, employing unified test papers and grading criteria. To address the comparability of academic performance across different grades, the average score of Chinese and Mathematics were standardized within each grade. Specifically, raw scores were first converted to grade-specific z-scores using Formula 1. These z-scores were subsequently transformed to T-scores using the linear transformation shown in Formula 2^19^. Children’s academic performance was assessed using T-scores.

Formula 1: $\:{z_{ig}} = \frac{{{X_{ig}} - {M_g}}}{{S{D_g}}}$; Formula 2: $\:{T_{ig}} = 50 + 10 \times {z_{ig}}$; i, individual student; g, grade level; X, raw score; M, grade-specific mean; SD, grade-specific standard deviation.

### Definition

Spherical equivalence (SE) was defined as the sum of the sphere and half of the cylinder (measured as negative values). Myopia was defined as SE ≤ −0.50 D after cycloplegia, premyopia as SE between > −0.50 D and ≤ + 0.75 D, and hyperopia as SE > + 0.75 D^20^. In myopic children, myopia undercorrection was defined as PVA > 0.1 logMAR, which could be improved by at least two lines using subjective refraction by increasing the minus lenses correction^21^. The myopia undercorrection rate is the proportion of children with undercorrected myopia among myopic children. Astigmatism was quantified as the cylinder power (negative notation) obtained from pre-cycloplegic auto-refraction and analyzed using absolute values. Anisometropia was defined as cycloplegic SE difference ≥ 1.00 D between eyes.

### Statistical analysis

Data from right eyes were analyzed using IBM SPSS Statistics (Version 25.0; IBM Corp., Armonk, NY, USA, 2017). Variables data were described as mean ± standard deviation (M ± SD), and attributes data were expressed as cases/percentage [N (%)]. The Shapiro-Wilk test was performed to assess the normality of the data. An independent two-sample T-test or one-way Analysis of Variance (ANOVA) was used to compare the differences between groups for continuous data, and the Least Significant Difference (LSD) method was used for one-way ANOVA post hoc tests. A Chi-square test was performed to compare the distribution of categorical variables between groups. Spearman’s correlation analysis was used to compare the correlations between groups for continuous data. T-scores of average academic performance across subjects were considered the primary dependent variable in constructing a Generalized Linear Model (GLM) to analyze the factors affecting academic performance. Variance Inflation Factor (VIF) diagnostics were performed to test the independent variables for multicollinearity. Variables demonstrating statistically significant associations (*P* < 0.05) in the univariate analysis and exhibiting VIF < 2 were included in the multivariate analysis. The β-values of the relevant factors and their 95% confidence interval (CI) were calculated. All statistical tests were two-sided and *P* < 0.05 was considered statistically significant.

## Results

### Demographic information

Of the 1,835 children, 1,766 (96.2%) were included in the analysis, as shown in Fig. 1. The average age of the children was 8.98 ± 1.23 years, with 52.2% (922/1,766) being boys. Among the participating children, 26.1% (461/1,766) had myopia, 31.3% (552/1,766) had hyperopia, and 51.0% (235/461) of the myopic children had undercorrected myopia. Table 1 shows the baseline ocular characteristics of the children. Table 2 shows the relationships between relevant factors and scores in Chinese, mathematics, and the average. In the Spearman’s correlation analysis, both AA (*r* = 0.10, *P* = < 0.001) and AF (*r* = 0.08, *P* = 0.002) were significantly correlated with Chinese scores. For mathematics scores, AA showed no significant correlation (*r* = 0.05, *P* = 0.053), while AF was significantly correlated (*r* = 0.08, *P* = 0.001).

**Table 1:** Baseline ocular characteristics of children.

Cycloplegic SE (D)	All (*N* = 1,766)	Hyperopia (*N* = 552)	Premyopia (*N* = 753)	Myopia (*N* = 461)
	0.08 ± 1.49	1.44 ± 0.85	0.28 ± 0.34	−1.18 ± 1.16
PVA (logMAR)	0.06 ± 0.13	0.01 ± 0.04	0.02 ± 0.06	0.19 ± 0.18
Astigmatism (D)	0.44 ± 0.51	0.39 ± 0.46	0.42 ± 0.45	0.55 ± 0.64
Binocular AF (cpm)	10.21 ± 4.45	10.51 ± 4.61	10.18 ± 4.16	9.90 ± 4.70
Binocular AA (D)	17.14 ± 3.67	17.63 ± 3.31	17.40 ± 3.56	16.11 ± 4.05
Anisometropia	161 (9.1%)	37(5.6%)	42(6.7%)	82(17.8%)

N: Number; M: Mean; SD: Standard deviation; SE: Spherical equivalence; PVA: Presenting visual acuity; AF: Accommodative facility; AA: Amplitude of accommodation; logMAR: Logarithm of the minimum angle of resolution; D: Diopter; cpm, Cycles per minute.

**Table 2:** Relationships between relevant factors and Chinese, mathematics and average academic performance (*N* = 1,766).

Age (Y)	*N* (%) or M ± SD	Chinese T-score (M ± SD)	*P*-value	Mathematics T-score (M ± SD)	*P*-value	Average T-score (M ± SD)	*P*-value
	8.98 ± 1.23	—	0.049^a^	—	0.298^a^	—	0.111^a^
School ID							
A	702(39.8%)	49.65 ± 12.06	0.264^b^	49.94 ± 10.16	0.850^b^	49.80 ± 10.86	0.507^b^
B	1064(60.2%)	50.23 ± 8.34		50.04 ± 9.88		50.13 ± 9.37	
Gender							
Boy	922 (52.2%)	48.69 ± 11.23	< 0.001^b^	49.41 ± 11.01	0.009^b^	49.00 ± 11.25	< 0.001^b^
Girl	844 (47.8%)	51.43 ± 8.21		50.64 ± 8.71		51.09 ± 8.27	
IQ							
tertile 1	558 (31.6%)	48.55 ± 11.61	< 0.001^c^	47.74 ± 11.84	< 0.001^c^	47.90 ± 11.88	< 0.001^c^
tertile 2	732 (41.4%)	49.98 ± 9.81		50.37 ± 9.39		50.22 ± 9.63	
tertile 3	476 (27.0%)	51.73 ± 7.70		52.07 ± 7.76		52.13 ± 7.29	
Refractive status							
Hyperopia	552 (31.2%)	50.75 ± 10.48	0.40^c^	50.24 ± 10.33	0.589^c^	50.52 ± 10.52	0.173^c^
Premyopia	753 (42.6%)	49.97 ± 9.94		50.06 ± 9.33		50.03 ± 9.05	
Myopia	461 (26.1%)	49.15 ± 9.41		49.61 ± 10.61		49.34 ± 10.10	
Anisometropia							
No	1605 (90.9%)	50.15 ± 9.92	0.055^b^	50.00 ± 10.07	0.977^b^	50.08 ± 9.99	0.285^b^
Yes	161 (9.1%)	48.47 ± 10.57		49.98 ± 9.14		49.20 ± 10.02	
Cycloplegic SE (D)	0.08 ± 1.49	—	0.036^a^	—	0.020^a^	—	0.030^a^
PVA (logMAR)	0.06 ± 0.13	—	< 0.001^a^	—	0.001^a^	—	< 0.001^a^
Astigmatism (D)	0.44 ± 0.51	—	0.001^a^	—	0.001^a^	—	< 0.001^a^
Binocular AF (cpm)	10.21 ± 4.45	—	0.002^a^	—	0.001^a^	—	< 0.001^a^
Binocular AA (D)	17.14 ± 3.67	—	< 0.001^a^	—	0.053^a^	—	0.001^a^

a: Spearman’s correlation; b: Independent Two-sample T-test; c: ANOVA. N: Number; M: Mean; SD: Standard deviation; SE: Spherical equivalence; PVA: Presenting visual acuity; AF: Accommodative facility; AA: Amplitude of accommodation; Y: Years; logMAR: Logarithm of the minimum angle of resolution; D: Diopter; cpm, Cycles per minute.

**Fig. 2:**
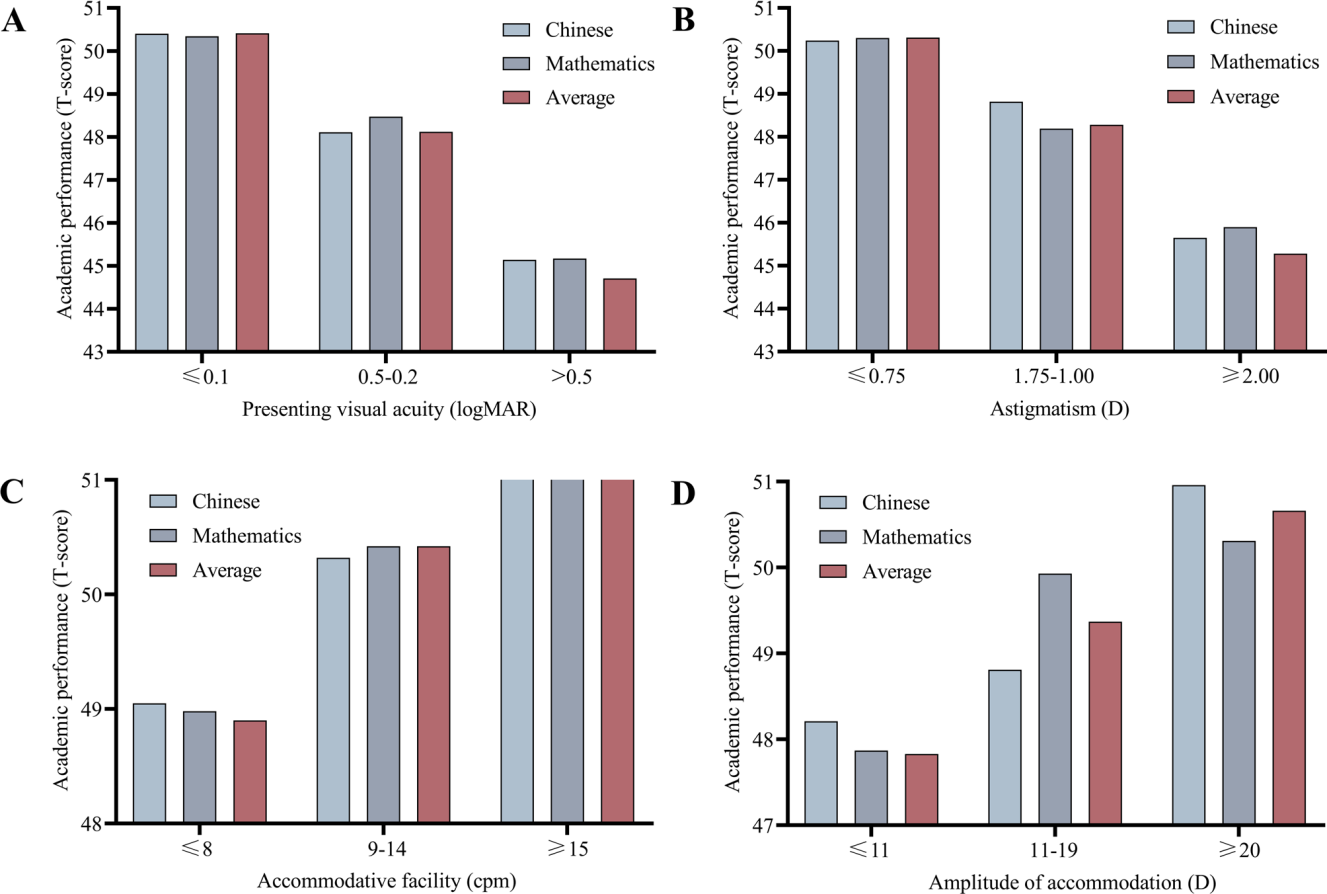
Distribution of children’s academic performance after stratification by PVA (**A**), Astigmatism (**B**), AF (**C**), and AA (**D**). logMAR: Logarithm of the minimum angle of resolution. D: Diopter; cpm: Cycles per minute.

**Fig. 3:**
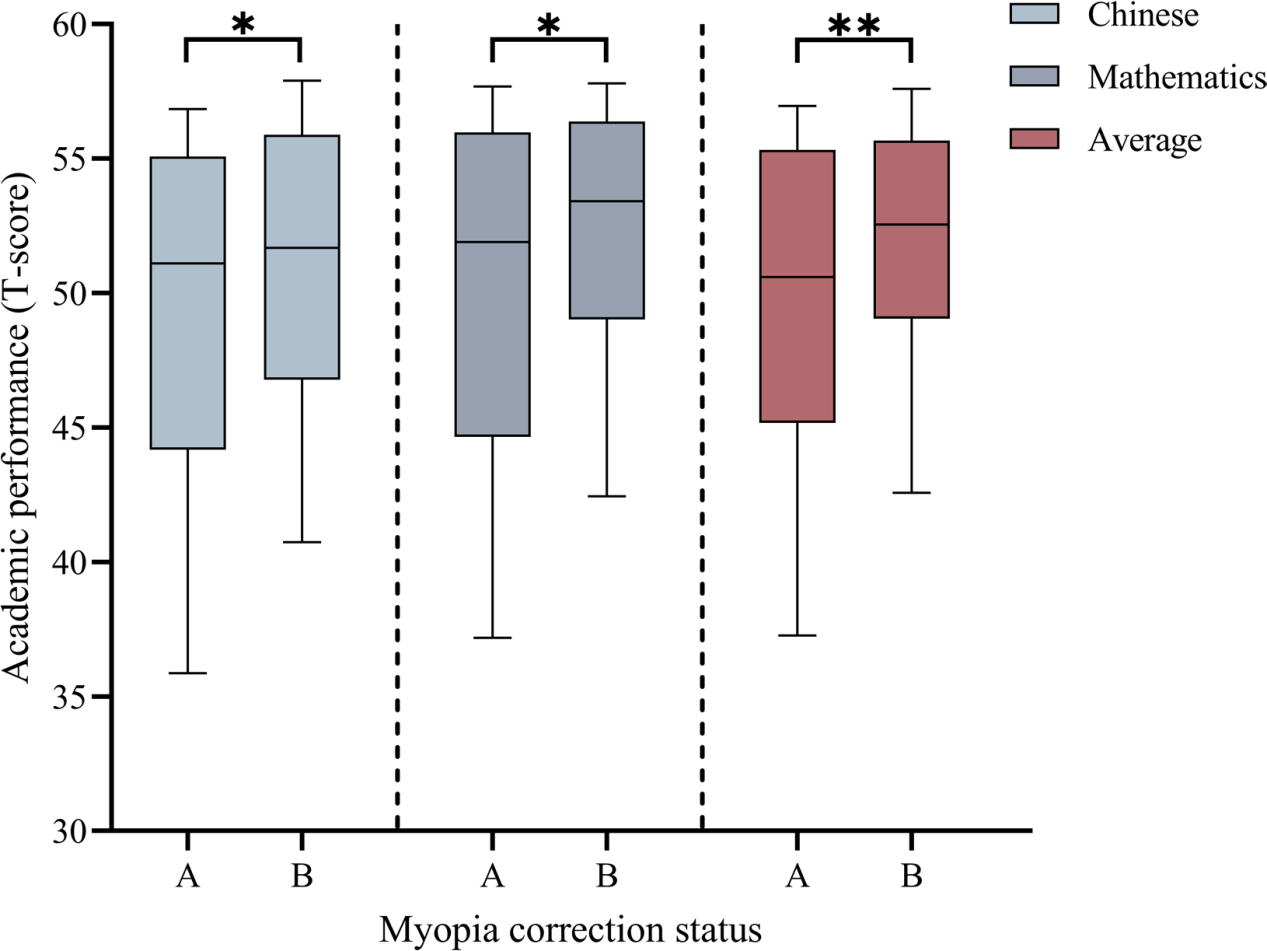
Comparing the academic performance of children with different myopia correction statuses. *: *P* < 0.05. **: *P* < 0.01. (**A**) Undercorrection group. (**B**) Full correction group.

Figure 2 shows that children with better PVA, less astigmatism, and higher binocular AF and binocular AA scores had better academic performance (*P* < 0.05). Among myopic children, those with undercorrected myopia had significantly poorer Chinese scores (*P* = 0.010), Mathematics scores (*P* = 0.010), and average scores (*P* = 0.004) than those with full correction (Fig. 3).

### Correlation analysis between visual factors and children’s academic performance

The multivariate GLM analysis (Table 3) showed that females (β = 2.30, 95% *CI*: 1.39 to 3.21; *P* < 0.001), IQ (tertile 1 vs. tertile 2, β = 2.40, 95% *CI*: 1.34 to 3.47; *P* < 0.001; tertile 1 vs. tertile 3, β = 4.27, 95% *CI*: 3.08 to 5.45; *P* < 0.001), binocular AF (β = 0.16, 95% *CI*: 0.06 to 0.26; *P* = 0.002) and binocular AA (β = 0.18, 95% *CI*: 0.05 to 0.30; *P* = 0.006) were significantly and positively correlated with academic performance. In contrast, PVA (β = −7.03, 95% *CI*: −10.63 to −3.43; *P* < 0.001) and astigmatism (β = −1.44, 95% *CI*: −2.33 to −0.55; *P* = 0.001) were significantly and negatively correlated with academic performance.

**Table 3:** Univariate and multivariate analysis of generalized linear models to assess the relationship between academic performance and influencing factors (*N* = 1,766).

	*N* (%) or M ± SD	Univariate analysis			Multivariate analysis		
		β	95% CI	*P*-value	β	95% CI	*P*-value
Age (Y)	8.98 ± 1.24	0.31	−0.07 to 0.68	0.111			
School ID							
A	702(39.8%)	0^a^					
B	1064(60.2%)	0.33	−0.62 to 1.28	0.494			
Gender							
Boy	922 (52.2%)	0^a^			0^a^		
Girl	844 (47.8%)	2.09	1.17 to 3.02	< 0.001	2.30	1.39 to 3.21	< 0.001
IQ							
tertile 1	558 (31.6%)	0^a^			0^a^		
tertile 2	732 (41.4%)	2.32	1.24 to 3.41	< 0.001	2.40	1.34 to 3.47	< 0.001
tertile 3	476 (27.0%)	4.22	3.02 to 5.43	< 0.001	4.27	3.08 to 5.45	< 0.001
Refractive status							
Hyperopia	552 (31.3%)	0.50	−0.60 to 1.59	0.375			
Premyopia	753 (42.6%)	0^a^					
Myopia	461 (26.1%)	−0.69	−1.84 to 0.47	0.246			
Anisometropia							
No	1,605 (90.9%)	0^a^					
Yes	161 (9.1%)	−0.88	−2.50 to 0.74	0.285			
Cycloplegic SE (D)	0.08 ± 1.49	0.20	−0.11 to 0.52	0.203			
PVA (logMAR)	0.06 ± 0.13	−7.99	−11.59 to −4.38	< 0.001	−7.03	−10.63 to −3.43	< 0.001
Astigmatism (D)	0.44 ± 0.51	−1.68	−2.59 to −0.77	< 0.001	−1.44	−2.33 to −0.55	0.001
Binocular AF (cpm)	10.21 ± 4.45	0.19	0.08 to 0.29	< 0.001	0.16	0.06 to 0.26	0.002
Binocular AA (D)	17.14 ± 3.67	0.21	0.08 to 0.34	0.001	0.18	0.05 to 0.30	0.006

N: Number; M: Mean; SD: Standard deviation; SE: Spherical equivalence; PVA: Presenting visual acuity; AF: Accommodative facility; AA: Amplitude of accommodation; Y: Years; logMAR: Logarithm of the minimum angle of resolution; D: Diopter; cpm: Cycles per minute; CI: Confidence interval; 0^a^: Reference group.

### Relationship between myopia correction status and academic performance

Subgroup analyses were performed after excluding children with premyopia and hyperopia. The myopic children were classified into a full correction group and an undercorrection group based on their PVA. Among them, the full correction group comprised 53.1% males (*n* = 120) and 46.9% females (*n* = 106), while the undercorrection group consisted of 42.6% males (*n* = 100) and 57.4% females (*n* = 135). The difference in gender distribution between the two groups was statistically significant (χ^2^ = 5.13, *P* = 0.023). Multifactorial GLM analysis (Table 4) showed that myopia undercorrection (β = −2.88, 95% *CI*: −4.68 to −1.09; *P* = 0.002) was significantly and negatively correlated with academic performance after adjusting for gender, IQ, and binocular AA.

**Table 4:** Univariate and multivariate analysis of generalized linear models to assess the relationship between academic performance and influencing factors in myopic children (*N* = 461).

Factors	*N* (%) M ± SD	Univariate analysis			Multivariate analysis		
		β	95% CI	*P*-value	β	95% CI	*P*-value
Age (Y)	8.90 ± 1.20	−0.02	−0.79 to 0.75	0.952			
School ID							
A	183(39.7%)	0^a^					
B	278(60.3%)	−0.65	−2.53 to 1.23	0.497			
Gender							
Boy	220 (47.7%)	0^a^			0^a^		
Girl	241 (52.3%)	3.13	1.31 to 4.95	0.001	3.53	1.74 to 5.33	< 0.001
IQ							
tertile 1	129 (28.0%)	0^a^			0^a^		
tertile 2	203 (44.0%)	0.66	−1.55 to 2.88	0.558	0.88	−1.28 to 3.04	0.426
tertile 3	129 (28.0%)	2.49	0.04 to 4.94	0.046	2.86	0.47 to 5.24	0.019
Myopia correction status							
Full correction	226 (49.0%)	0^a^			0^a^		
Undercorrection	235 (51.0%)	−2.66	−4.49 to −0.84	0.004	−2.88	−4.68 to −1.09	0.002
Anisometropia							
No	379 (82.2%)	0^a^					
Yes	82 (17.8%)	0.64	−1.76 to 3.05	0.600			
Astigmatism (D)	0.55 ± 0.64	0.14	−1.31 to 1.59	0.849			
Binocular AF (cpm)	9.90 ± 4.70	0.16	−0.04 to 0.36	0.110			
Binocular AA (D)	16.11 ± 4.05	0.26	0.04 to 0.49	0.022	0.25	0.03 to 0.47	0.029

N: Number; M: Mean; SD: Standard deviation; SE: Spherical equivalence; PVA: Presenting visual acuity; AF: Accommodative facility; AA: Amplitude of accommodation; Y: Years; D: Diopter; cpm: Cycles per minute; CI: Confidence interval; 0^a^: Reference group.

## Discussion

This study found that PVA was an independent factor influencing academic performance (β = −7.03, *P* < 0.001), showing a significant negative correlation with academic performance, which is consistent with previous studies^5^. This may be explained by the difficulty children with poor PVA experience in clearly viewing content on blackboards or projection screens. Additionally, the results showed that astigmatism was significantly negatively correlated with academic performance (β = −1.44, *P* = 0.001). Higher magnitudes of astigmatism can impact children’s academic performance by reducing PVA^22^ and hindering reading fluency^23^.

Abnormalities in AF and AA often cause symptoms such as headache, diplopia, blurred vision, and visual fatigue^24^, affecting children’s attention, reading, writing, and other learning-related activities, potentially leading to dyslexia^25,26^ and poor academic performance. When a child exhibits these symptoms, parents and teachers should consider that the child may have binocular vision dysfunction that must be addressed. Visual therapy improves binocular anomalies in children with learning disability^27^. Children with AF and AA anomalies can also improve their academic performance through such interventions^28^. Interestingly, in this study, AA (β = 0.18) showed a slightly stronger correlation with academic performance than AF (β = 0.16). This may be because AA represents the maximum accommodative reserve that can be mobilized during near work, which is crucial for prolonged reading and writing tasks in the classroom, particularly in the predominantly near-work environment of primary schools. Furthermore, AA showed a slightly higher correlation with Chinese scores (*r* = 0.10, *P* = < 0.001) than AF (*r* = 0.08, *P* = 0.002), while AF was significantly correlated with mathematics scores (*r* = 0.08, *P* = 0.001) and AA showed no significant correlation with mathematics scores (*r* = 0.05, *P* = 0.053). This suggests that different academic subjects may impose distinct demands on visual function. Specifically, Chinese learning may rely more on accommodative reserve, whereas mathematics may require greater accommodative flexibility.

Myopia undercorrection (β = −2.88, *P* = 0.002) has a negative effect on academic performance, which may be related to low PVA and decreased accommodation function in children with undercorrected myopia. Ma et al.^29^ and Latif et al.^30^ reported that academic performance significantly improved after children received full corrections. Furthermore, myopia undercorrection may result in increased axial elongation and faster myopia progression^31^. If not corrected in time, the extent of undercorrection will continue to worsen, and PVA will further decline. Therefore, myopic children need to undergo regular follow-up visits and replace their spectacles timely according to the ophthalmologist’s recommendation to maintain a good quality of vision. Consistent with previous studies^5,8^, we did not find an association between children’s academic performance and refractive status or cycloplegic SE. However, we recommend that children maintain healthy eye habits, such as increasing outdoor activities and exercise, ensuring adequate sleep, and reducing screen time. These behaviors are associated with a lower risk of myopia onset and progression, as well as a reduced likelihood of undercorrection. They are also linked to better academic performance in observational studies^32–34^.

Our findings highlight a potential for synergistic benefits between strategies aimed at myopia control and those supporting educational outcomes in children. In the future, it may be worth considering incorporating visual function assessment such as AA and AF into routine vision screening to establish a more comprehensive visual health evaluation system. Therefore, within a home-school collaborative framework, it is reasonable to recommend cultivating proper eye-use behaviors, as this approach aligns with both visual health and academic development goals.

This school-based study has several strengths, including a large sample size and high participation rate, enhancing the research results’ reliability. We adjusted for a broader range of confounding factors, extending beyond visual acuity to include other potential vision-related factors. Additionally, we considered the effect of myopia correction status on children’s academic performance. However, this study has some limitations that must be noted. First, it used primary school children as the study population, limiting the generalizability of the results to children in other academic segments. Second, although we standardized academic scores by grade, the lack of cross-grade scores limits the direct comparability of results across different grades. Third, the children in this study were native Chinese speakers, and the results may differ for children in countries and regions where English or Latin is used. Finally, the limitations of the cross-sectional design prevented us from determining the causal relationship between academic performance and visual factors, although we comprehensively adjusted for confounders. Further longitudinal investigations spanning wider age groups and varied educational environments are required to better understand the relationship between visual factors and academic performance.

## Conclusion

Academic performance is a critical indicator of children’s learning progress and greatly concerns parents and educators. Visual factors, such as visual acuity, astigmatism, ocular accommodation function, and correction status, can significantly influence children’s academic performance. Parents and schools should pay more attention to children’s visual health for early prevention and intervention so that they can maintain a good visual status during the learning process. For myopic children, regular follow-ups should be conducted, scientific and reasonable correction strategies should be formulated, and accurate and complete corrections should be administered promptly.

## Data Availability

The datasets analysed during the current study are available from the corresponding author on reasonable request.
